# Estimation of Economic Indicator Announced by Government From Social Big Data

**DOI:** 10.3390/e20110852

**Published:** 2018-11-06

**Authors:** Kenta Yamada, Hideki Takayasu, Misako Takayasu

**Affiliations:** 1 Institute of Innovative Research, Tokyo Institute of Technology, 4259, Nagatsuta-cho, Yokohama 226-8502, Japan; 2 Sony Computer Science Laboratories, 3-14-13, Higashi-Gotanda, Shinagawa-ku, Tokyo 141-0022, Japan; 3 Department of Mathematical and Computing Science, School of Computing, Tokyo Institute of Technology, 4259, Nagatsuta-cho, Yokohama 226-8502, Japan

**Keywords:** big blog data, economic index, robust variable selection, regression analysis

## Abstract

We introduce a systematic method to estimate an economic indicator from the Japanese government by analyzing big Japanese blog data. Explanatory variables are monthly word frequencies. We adopt 1352 words in the section of economics and industry of the Nikkei thesaurus for each candidate word to illustrate the economic index. From this large volume of words, our method automatically selects the words which have strong correlation with the economic indicator and resolves some difficulties in statistics such as the spurious correlation and overfitting. As a result, our model reasonably illustrates the real economy index. The announcement of an economic index from government usually has a time lag, while our proposed method can be real time.

## 1. Introduction

By analyzing social big data such as blogs, twitter, etc., many statistical properties of human behaviors are uncovered scientifically. Typical examples are power law growth and relaxation of social interests around social events such as Christmas [1,2], spreading properties of false rumors and fake news [3,4], relationships between stock prices and social data [5,6], predicting consumer behavior with Web search [7] and forecasting macroeconomic index with Google Trends [8]. It is a big challenge for mathematical scientists to establish data analysis methods and propose mathematical models to describe and predict the phenomena. Currently, much attention is being paid to the topic of finding significant correlations between phenomena in the real world and in cyberspace.

In this paper, we examine the relation between a composite index (CI) and big blog data. The CI that characterizes the Japanese economy is announced by the Japanese government every month. Although many people want to know the latest economic condition quickly, it takes a few months to calculate CI values based on the observed quantities in many fields such as production, inventory, investment, employment, consumption, business management, finance, prices and services. In this paper, we show that the value of CI can be roughly estimated by the appearance of carefully chosen words in cyberspace. As the analysis using data in the cyberspace can be conducted in real time at lowers costs, it is possible that CI could be approximated by applying this new method for quick announcement.

We expect that online textual data such as blogs and Twitter are related to economic indices. Recently, many diverse people have begun to use social media and the data posted on such sites immediately reflect social events and human activities. For example, earthquakes and typhoons can be detected quickly and accurately by using Twitter data [9]. It detected 96% of earthquakes of Japan Meteorological Agency (JMA) seismic intensity scale 3 or higher and announced more quickly than the announcements that were broadcast by the JMA. In addition, power law growth and relaxation were observed in blog frequency with words such as “April Fool” or “Christmas” around these social events. The future frequency of such words could be predicted by the properties [1].

Nowcasting and forecasting based on social big data are effective because social data in cyberspace reflect public opinion and behavior in physical space. However, there are difficulties because of the large volume of data. For example, in the case of a huge number of candidates, such as 10,000 variables, if we assign the threshold of *p*-value as 0.01, then approximately 100 variables would remain, even if these candidates followed independent randomness.

We also have to pay attention to the following points in conducting correlation and regression analyses. First, the Pearson correlation coefficient is defined as follows:

(1) $$\rho =\langle Z_{x}\cdot Z_{y}\rangle ,$$

where $\langle Z\rangle$ represents an average of *Z* and ρ has a value between +1 and −1. If ρ>0, the correlation is positive; conversely, if ρ<0, the correlation is negative. $Z_{x}$ and $Z_{y}$ are *Z*-scores defined as:

(2) $$\begin{array}{c} Z_{x}=\frac{x-\langle x\rangle }{\sigma_{x}},\;Z_{y}=\frac{y-\langle y\rangle }{\sigma_{y}}, \end{array}$$

which are normalized to an average 0 and standard deviation 1. The Pearson correlation coefficient is a useful tool in describing the relationship between two variables; however, when we apply it, we should be aware of the following assumptions.

- Two variables follow a linear relationship.
- Averages and standard deviations are well-defined.

Namely, if two variables do not follow a linear relation, or if variables (*x*, *y*) include outliers that significantly affect the correlation coefficient, we cannot trust the coefficient. In addition, in cases where the distributions of *x* or *y* follow power law distributions, $\left(P\left(x\right)\propto x^{-\beta },\;0<\beta \leq 2\right)$, the standard deviation tends to diverge when the number of samples increases. Therefore, we need to consider that the Pearson correlation coefficient is meaningless in this context.

Second, we focus on the possibility that a correlation may be spurious. For instance, we assume three variables, e.g., temperature, and the sales of ice cream and soft drinks. Calculating correlations among these values, we probably find positive correlations. We consider when the temperature rises, people tend to buy more ice cream and soft drinks to provide relief from the heat; however, it is not reasonable to assume that the sales of soft drinks are increasing because the sales of ice cream are rising. In this case, we find the positive correlation between sales of ice cream and soft drinks. However, this could happen even if there is no relation among these variables, in which case we call the correlation a spurious correlation. When we detect a strong correlation, we are inclined to consider the existence of a linkage that includes a causal relationship, which often leads to an incorrect result. Therefore, when we deal with multiple variables, we need to introduce a process which excludes spurious correlations.

Third, when we apply regression analysis, we must pay attention to the following points:

- Overfitting
- Spurious regression
- Multicollinearity

Overfitting is the case where we take many variables to reproduce details of training data, however, the model does not have the same performance for the test data, which are not used in the training. If we run regression analysis to non-stationary time series, we often have a large coefficient of determination, which is called a spurious regression [10,11]. Moreover, if more than two explanatory variables are a strongly correlated, the relationships between the explanatory variables are not independent. Therefore, optimal regression coefficients are not uniquely obtained, which is called multicollinearity. In this case, the reliability of the regression coefficients is remarkably reduced.

In cases where we handle multivariate variables with non-normal distribution and non-stationary dynamics, it is important to carefully check their appropriateness for the Pearson correlation coefficient and regression analysis. In the next section, we introduce a method which removes these difficulties and estimates the economic index using big blog data.

## 2. Development of a New Economic Index Based on a Comprehensive Search

In this section, we introduce a new method that systematically finds words that are correlated with respect to an economic index, and it reconstructs the index by using a regression formulation with the frequency of selected words.

**Figure 1:**
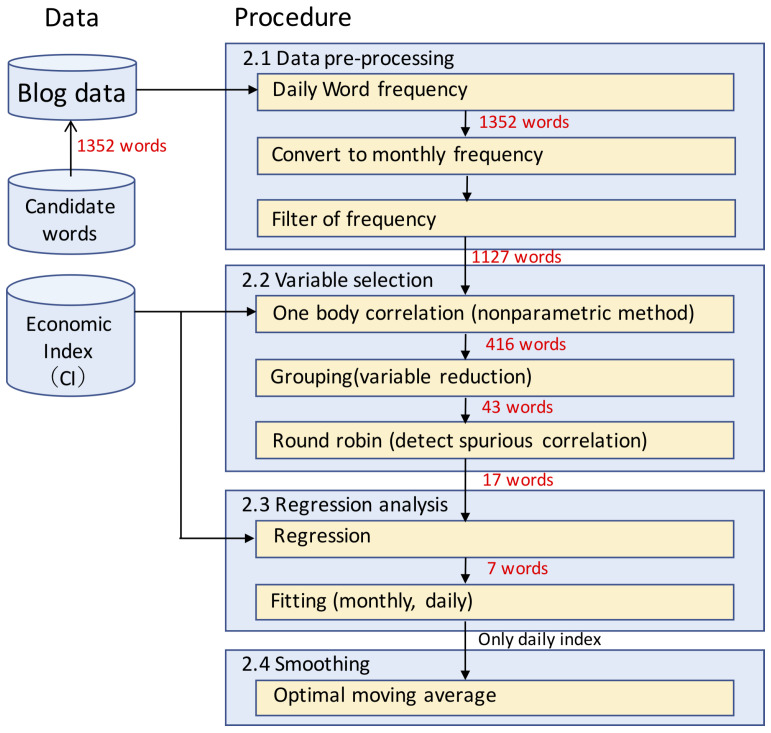
Flowchart of estimating an economic index from many word frequencies based on comprehensive search.

The method, which resolves all the problems mentioned in the Introduction, consists of the following four steps, as shown in Figure 1: data pre-processing, variable selection, regression analysis and smoothing.

In this analysis, we used three types of data: blogs, dictionary of candidate words and the time series of an economic index. As for blogs, we used “Kuchikomi@kakaricho” (http://kakaricho.jp) to obtain the daily frequencies of words, which covers about 1.8 billion blogs from 1 January 2007 to 31 October 2015. We adopted 1352 words listed in the economics and industry section of the Nikkei thesaurus (http://t21.nikkei.co.jp/public/help/contract/price/20/thesaurus/field_B.html) to illustrate the economic index. In this paper, we focus on the index of business conditions, especially Composite Index (CI) coincidence, which is announced monthly by the Japanese government. The CI provides a quantitative description of the Japanese economy.

### 2.1. Data Pre-Processing

We analyzed the daily frequency of each of the 1352 words from 1 January 2007, which is described by $r_{i}\left(t_{\mathrm{day}}\right)$ where *i* is an index of words and $t_{\mathrm{day}}$ is daily time. Here, $r_{i}\left(t_{\mathrm{day}}\right)$ is defined as follows:

(3) $$r_{i}\left(t_{\mathrm{day}}\right)=\frac{w_{i}\left(t_{\mathrm{day}}\right)}{W(t_{\mathrm{day}})},$$

where $w_{i}\left(t_{\mathrm{day}}\right)$ is the number of blog entries including the *i*th key word, and $W(t_{\mathrm{day}})$ is the total number of blogs on $t_{\mathrm{day}}$. Namely, $r_{i}\left(t_{\mathrm{day}}\right)$ is a value by normalizing $w_{i}\left(t_{\mathrm{day}}\right)$ with $W(t_{\mathrm{day}})$ to remove the effects such as trend of the entire blog and the day of the week effect because people tend to write blogs on the weekend. We also calculate monthly average frequency as,

(4) $$r_{i}\left(t_{\mathrm{month}}\right)=\frac{1}{m}\sum_{k=1}^{m}r_{i}\left(t_{\mathrm{day}}\right),$$

to match with the economic index intervals which is announced monthly. Here, *m* is the number of days in one month: 28, 29, 30 or 31. We excluded the words with 0 in frequency over 10 months to remove very low frequency words. Thus, 1127 candidate words remained.

### 2.2. Variable Selection

#### 2.2.1. Extraction of Words by One-Body Correlation

**Figure 2:**
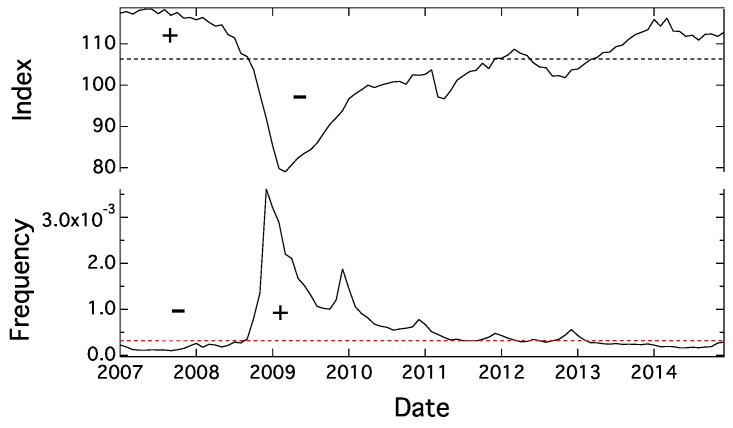
The upper chart shows the Composite Index (CI) coincidence and the lower chart is monthly frequency of “hukeiki” (recession) from January 2007 to December 2014. Broken lines represent the median.

First, we introduced an algorithm that chooses the words with similar patterns and trends in the time series of frequency. Specifically, as shown in Figure 2, we gave a “+” sign if the value is greater than the median of the time series, while we used a “−” sign if the value is smaller. There were four patterns with respect to both values of CI and word frequency, which are greater or smaller than the median: (CI, word frequency) = $\left\{(+,+),\;(+,-),\;(-,+),\;(-,-)\right\}$. If we find more frequency of (+,+) and (−,−) than (+,−) and (−,+), CI and word frequency have positive correlation. Alternatively, if (+,−) and (−,+) appear more often than (+,+) and (−,−), the two time series have negative correlation. Using the number of appearances of (+,+),(+,−),(−,+),(−,−) as a=♯(+,+),b=♯(+,−),c=♯(−,+),d=♯(−,−), we define *p* as no correlation degree.

(5) $$p=\sum_{P\leq P_{0}}P,\;P_{0}=\frac{(a+b)!(c+d)!(a+c)!(b+d)!}{n!a!b!c!d!},\;n=a+b+c+d$$

This definition is the same as that of probability in Fisher’s exact test. In this analysis, we imposed the restriction of dividing the median, and therefore the probability *p* does not have exactly the same meaning as the original *p* in Fisher’s exact test. In this analysis, we considered that, if $p<10^{-3}$, the variables are regarded as sufficiently correlated. As a result, we found 416 words that have small *p*-values that show strong correlations with CI. The Top 4 words with the smallest *p*-values are shown in Table 1.

#### 2.2.2. Grouping

The words selected in the previous method are likely to have very similar patterns and trends. If we apply a regression analysis using all the words, we have the multicollinearity problem and overfitting problem mentioned in the Introduction. To solve these problems, we categorized the words into some groups which have similar patterns and trends.

First, we assigned a number (1,2,3,⋯) in ascending order of the no correlation index, *p* (in descending order of having strong correlation). Next, we calculated the value *p* defined in Equation (5) for *i*th and *j*th words (i<j) as well as shown in the extraction of words by one-body correlation, and, if the value *p* was smaller than the threshold value ($10^{-6}$), the time series of frequencies of two words was judged as very similar and the *j*th word was classified into the *i*th word’s group. By applying this algorithm to pairs in descending order, i.e., 1st–2nd, 1st–3rd, ⋯, 2nd–3rd, 2nd–4th, etc., we formed a group of words.

**Figure 3:**
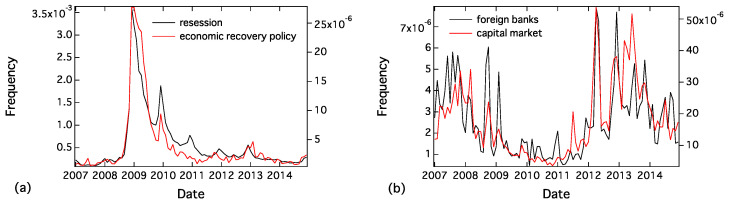
Two typical examples of grouped words: (**a**) recession and economic recovery policy; and (**b**) foreign banks and capital market.

Figure 3 shows an example of the grouping results. We can observe the time series of pairs: for example, “hukeiki” (recession) and “hukyou taisaku” (economic recovery policy), and “gaikoku ginkou” (foreign banks) and “shihon shizyou” (capital market) are comparable. After the grouping process, we classified the 416 words into 43 groups whose representative words have the lowest *p* in the group.

#### 2.2.3. Round Robin (Detection of Spurious Correlation)

Based on Bayesian analysis for Dirichlet distribution [12], we defined a probability in which both frequency of *i*th words and CI have a larger value than the median,

(6) $$q_{i}\left(a,b\right)=\frac{a+2r}{a+b+2},$$

where a=♯(+,+) and b=♯(+,−), as shown in Section 2.2.1, and *r* is the probability that CI has a larger value than its median estimated from whole data:

(7) $$r=\frac{a+c}{a+b+c+d}.$$

where c=♯(−,+) and d=♯(−,−). We also define corresponding standard deviation as

(8) $$\sigma_{i}\left(a,b\right)=\frac{1}{a+b+2}\sqrt{\frac{\left(a+2r)(b+2-2r\right)}{a+b+3}}.$$

In the cases where the frequency of the *i*th word has a lower probability than the median, we defined the following values as well as the frequency that has a larger value than the median:

(9) $$\begin{array}{ccc} q_{\bar{i}}\left(c,d\right) & = & \frac{c+2r}{c+d+2}, \end{array}$$

(10) $$\begin{array}{ccc} \sigma_{\bar{i}}\left(c,d\right) & = & \frac{1}{c+d+2}\sqrt{\frac{\left(c+2r)(d+2-2r\right)}{c+d+3}}. \end{array}$$

To introduce conditional probabilities with respect to the *j*th word, we defined the following quantities, $a^{\prime }=♯\left(+,+\right),\;b^{\prime }=♯\left(+,-\right),\;c^{\prime }=♯\left(-,+\right),\;d^{\prime }=♯\left(-,-\right)$ as the numbers that CI and the frequency of the *i*th word take a greater value than each median under the condition where the *j*th word has a bigger value compared to its median in the same month. The probability and standard deviation in the condition are defined below:

(11) $$\begin{array}{ccc} q_{i|j}\left(a^{\prime },b^{\prime }\right) & = & \frac{a^{\prime }+2r}{a^{\prime }+b^{\prime }+2}, \end{array}$$

(12) $$\begin{array}{ccc} q_{\bar{i}|j}\left(c^{\prime },d^{\prime }\right) & = & \frac{c^{\prime }+2r}{c^{\prime }+d^{\prime }+2}, \end{array}$$

(13) $$\begin{array}{ccc} \sigma_{i|j}\left(a^{\prime },b^{\prime }\right) & = & \frac{1}{a^{\prime }+b^{\prime }+2}\sqrt{\frac{\left(a^{\prime }+2r\right)\left(b^{\prime }+2-2r\right)}{a^{\prime }+b^{\prime }+3}}, \end{array}$$

(14) $$\begin{array}{ccc} \sigma_{\bar{i}|j}\left(c^{\prime },d^{\prime }\right) & = & \frac{1}{c^{\prime }+d^{\prime }+2}\sqrt{\frac{\left(c^{\prime }+2r\right)\left(d^{\prime }+2-2r\right)}{c^{\prime }+d^{\prime }+3}}. \end{array}$$

In addition to $a^{\prime },\;b^{\prime },\;c^{\prime }\;\mathrm{and}\;d^{\prime }$, we defined $a^{\prime \prime }=♯\left(+,+\right),\;b^{\prime \prime }=♯\left(+,-\right),\;c^{\prime \prime }=♯\left(-,+\right),\;d^{\prime \prime }=♯\left(-,-\right)$, which describe the numbers that both CI and the frequency of the *j*th word have a larger value than each median in the condition where the *i*th word has a bigger value compared to its median. We also defined $q_{i|\bar{j}}\left(a^{\prime \prime },b^{\prime \prime }\right)$, $q_{\bar{i}|\bar{j}}\left(a^{\prime \prime },b^{\prime \prime }\right)$, $\sigma_{i|\bar{j}}\left(a^{\prime \prime },b^{\prime \prime }\right)$, $\sigma_{\bar{i}|\bar{j}}\left(a^{\prime \prime },b^{\prime \prime }\right)$ in the same way as Equations (11)–(14).

Using these values, we defined the effectiveness of the *i*th word to illustrate CI, $k_{i|j}$, in a condition where the frequency of the *j*th word is bigger than the median:

(15) $$k_{i|j}=\max\left(\frac{q_{i|j}-q_{\bar{i}|j}}{\sigma_{i|j}},\frac{q_{i|j}-q_{\bar{i}|j}}{\sigma_{\bar{i}|j}}\right).$$

Similarly, we defined the following quantity with the condition that the frequency of the *j*th word is smaller than its median:

(16) $$k_{i|\bar{j}}=\max\left(\frac{q_{i|\bar{j}}-q_{\bar{i}|\bar{j}}}{\sigma_{i|\bar{j}}},\frac{q_{i|\bar{j}}-q_{\bar{i}|\bar{j}}}{\sigma_{\bar{i}|\bar{j}}}\right).$$

If these values, which were conditioned by the *j*th word, are small, the correlation between the *i*th word and CI is likely to be spurious correlation caused by the *j*th word. We introduced the following quantity, which characterizes the strength of the correlation between the *i*th word and CI taking into account the condition of the *j*th word.

(17) $$P\left(i:j\right)=k_{i|j}+k_{i|\bar{j}}.$$

Some round robin results of P(i:j) are shown in Table 2. If P(j:i) is smaller than P(i:j), we considered that the *j*th word has a spurious correlation caused by the *i*th word, because the correlation with a condition by the *i*th word against the *j*th word is smaller than a condition by the *j*th word against *i*th word. To evaluate the asymmetry, we defined an asymmetrical index:

(18) $$D\left(i:j\right)=\frac{P(i:j)-P(j:i)}{P(i:j)+P(j:i)}.$$

If D(i:j) has a larger value than a given threshold (−0.6 in this analysis), we judged that the *i*th word has a spurious correlation caused by the *j*th word. For example, regarding the pair “keiki taisaku” (economic measures) and “hukeiki” (recession),

(19) $$D\left(“keiki\;taisaku”\;(\mathrm{economic}\;\mathrm{measures}):“hukeiki”\;(\mathrm{recession})\right)=\frac{7.6-31.7}{7.6+31.7}=-0.61.$$

We regarded “keiki taisaku” (economic measures) as having spurious correlation against “hukeiki” (recession). Therefore, “keiki taisaku” (economic measures) was classified into the group of words which is represented by the word, “hukeiki” (recession).

After classifying the words, we computed the following sum of P(i:j)

(20) $$S\left(i\right)=\sum_{j}P\left(i:j\right).$$

In the regression analysis, we adopted, as an explanatory variable, the descending order of S(i).

#### 2.2.4. Visualization of Relationships Between Variables

**Figure 4:**
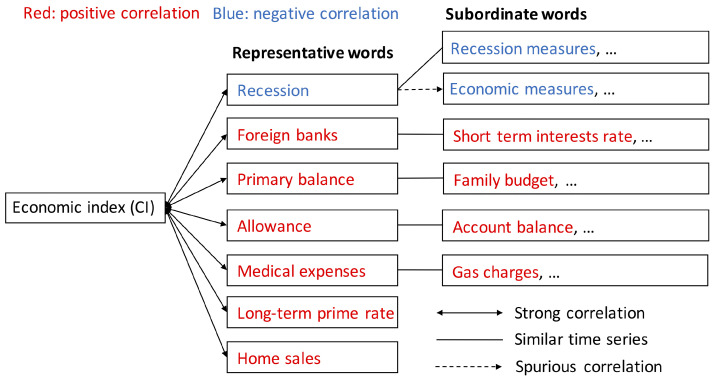
The relationships between variables: (i) economic index and words; and (ii) representative words and subordinate words as a result of analyses: extraction of words by one-body correlation, grouping and round robin (detection of spurious correlation).

To intuitively understand the results obtained in the previous steps, such as the extraction of words by the one-body correlation, grouping and round robin (detection of a spurious correlation), we show the relationships between words in Figure 4. In this figure, the solid line with both sides arrow indicates relationships with a strong correlation between CI and word frequency. These words passed the judgments: grouping and detection of spurious correlation. There are 17 representative words and, in this figure, we show the Top 7 words. The solid line without an arrow describes the words which are grouped in the representative words, and the broken line with an arrow indicates a spurious correlation.

### 2.3. Regression Analysis

We designed a model that reconstructs CI by conducting a regression analysis with frequency of words such as “hukeiki” (recession), “gaikoku ginkou” (foreign banks), etc., which were selected in the previous analysis. We applied this model to estimate the daily CI based on the daily frequency of words.

We performed a linear regression analysis using CI as a dependent variable and the frequency of words as explanatory variables:

(21) $$\tilde{y}=c_{0}+\sum_{k=1}^{m}c_{k}x_{k},$$

where $c_{0}$ is a constant and *m* means the number of representative words. We used top *m* words with respect to the order of S(i) in Equation (20). We then obtained coefficients $c_{k}$ with the minimum square error:

(22) $$\Delta y=\sum_{k}{\left(y_{k}-\tilde{y_{k}}\right)}^{2}$$

**Figure 5:**
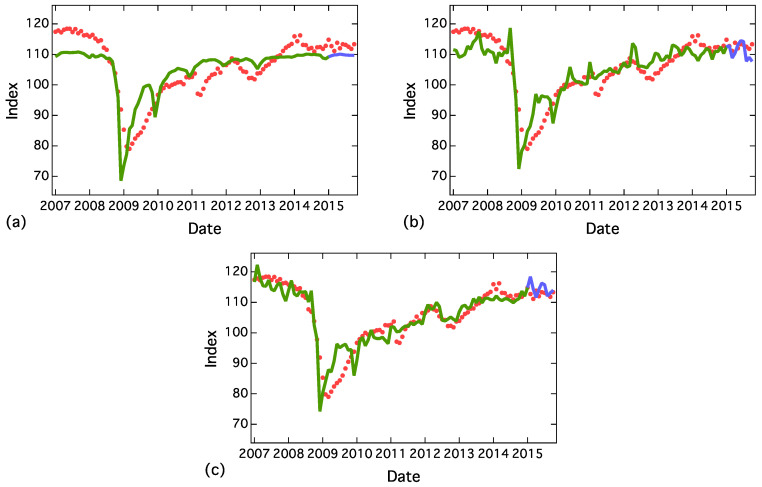
Regression results using the following word frequencies: (**a**) recession (one word), (**b**) recession, foreign banks and primary balance (three words), and (**c**) recession, foreign banks, primary balance, allowance, medical expenses, long-term prime rate and home sales (seven words). Training period was from January 2007 to December 2014 (green line) and test period was from January 2015 to October 2015 (blue line).

Figure 5 depicts the regression results using one, three and seven variables. In the case of using only one variable, “hukeiki” (recession), big jumps in 2008 and 2009 were reproduced, and, by adding other variables to our model, more details were reproduced. The regression coefficients in the case of 7 words are shown in Table 3. Besides the results of in-sample data, our model yielded reasonable results for the out-sample data from January to October 2015.

In the above variable selection analysis, we obtained 17 representative words from 1352 words. We then introduced the method that allowed us to systematically decide the appropriate number of explanatory variables. As discussed in the Introduction, we applied the regression analysis to non-stationary time series CI. To check for the spurious regression problem, we proposed a hypothesis testing which compared the frequency of words and an artificial time series which follows the random walk model.

**Figure 6:**
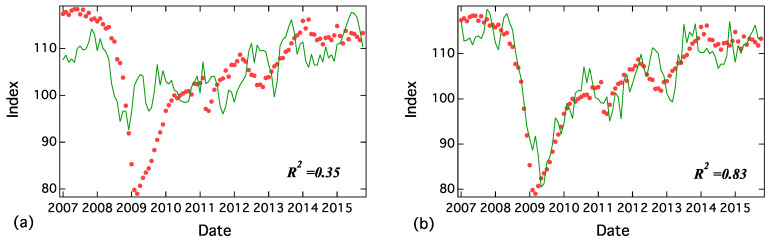
Regression results of CI by seven random walks. In the case of (**a**), $R^{2}=0.35$, and in the case of (**b**), $R^{2}=0.83$.

A random walk time series was generated by randomly sampled numbers from the same distribution with real time series. For example, an absolute value of differences (step size) of the first random walk was randomly chosen from the absolute value of the differences in “hukeiki” (recession) and we then gave positive or negative signs randomly. The *k*th random walks were produced using the absolute value of differences of the top *k*th word as well as the first one. Figure 6 shows the results of fitting CI by seven random walks. In Figure 6a, the fitting did not work well and $R^{2}$ is low, where $R^{2}$ is defined as follows:

(23) $$R^{2}=1-\frac{\sum_{i=1}^{N}{\left(y_{i}-\tilde{y_{i}}\right)}^{2}}{\sum_{i=1}^{N}{\left(y_{i}-\langle y\rangle \right)}^{2}}.$$

Here, *N* is the number of data. In Figure 6b, the random walk regression reconstructed the properties of CI with $R^{2}=0.83$. These result indicate that random walk sometimes reproduce non-stationary time series.

**Figure 7:**
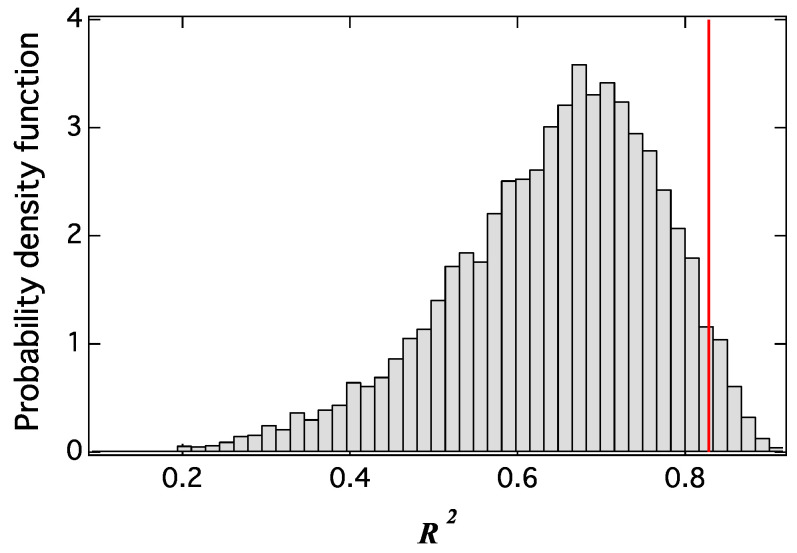
Probability density function of $R^{2}$ for 10,000 samples by the regression of seven random walks. The red line shows the result in Figure 5c.

Figure 7 shows the probability density function of $R^{2}$ for 10,000 samples by the regression of seven random walks. The red line in this figure shows the result in Figure 5c. The results in Figure 5c had a greater $R^{2}$ value compared to the random walks in most cases, and the probability of the random walks model having a higher $R^{2}$ was approximately 3%.

**Figure 8:**
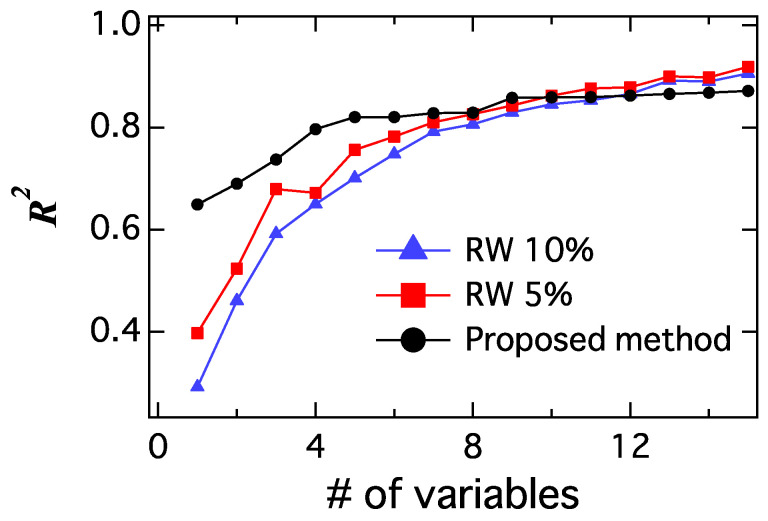
The relationship between the coefficient of determination ($R^{2}$) and the number of explanatory variables for the proposed model (black) and random walk model with Top 5 and 10 percentiles (red and blue).

Using the distribution of $R^{2}$ by the random walk regression, we performed a statistical test. The null hypothesis is the following: “The random walks regression is more effective than the regression by the time series of selected words with respect to $R^{2}$”. Figure 8 shows the relationships between the coefficient of determination ($R^{2}$), the number of explanatory variables in the proposed model, and the random walk model with the Top 5 and 10 percentiles. The values of the coefficient of determination increases with respect to the number of explanatory variables. In this case, the $R^{2}$ of the proposed method was higher than the case of the Top 5 percentile of the random walk model until seven variables. This means that the null hypothesis was rejected with a significant level of 5% and proposed model was better than the random walk model statistically. However, in the case of more than eight variables, the null hypothesis with a significance level of 5% was not rejected. Therefore, we considered the proposed method with seven variables appropriate. The number of explanatory variables is determined as

(24) $$m=\underset{k}{\arg\;\min}\left(R_{k}^{2}\left(\mathrm{proposed}\;\mathrm{method}\right)<R_{k}^{2}\left(\mathrm{RW}5\%\right)\right)-1,$$

where $R_{k}^{2}$ (proposed method) shows the coefficient of determination of the proposed method with the top *k* explanatory variables and $R_{k}^{2}$ (RW 5%) is the coefficient of determination of the random walk model in the top 5% percentile.

### 2.4. Daily Index and Smoothing

The daily index $I(t_{\mathrm{day}})$ can be calculated by the regression coefficients and daily frequency of words ($r_{k}\left(t_{\mathrm{day}}\right)$),

(25) $$I\left(t_{\mathrm{day}}\right)=c_{0}+\sum_{k=1}^{m}c_{k}r_{k}\left(t\right).$$

**Figure 9:**
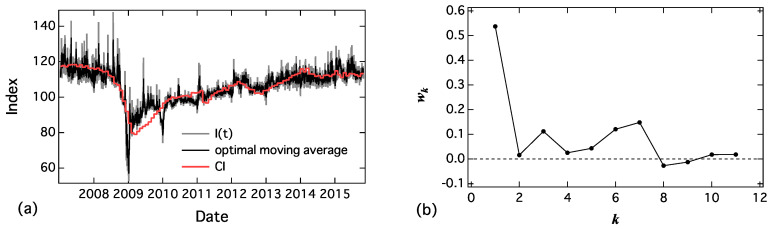
(**a**) Daily index, $I(t_{\mathrm{day}})$, calculated by Equation (25) and optimal moving average defined by Equation (26); and (**b**) estimated optimal weight $w_{k}$ in Equation (26).

$I(t_{\mathrm{day}})$, as shown in Figure 9a, suddenly increased in response to the news related to the words. To reduce this effect, we applied the optimal moving average [13]. The optimal moving average ($\bar{I}\left(t\right)$) of $I(t_{\mathrm{day}})$ is calculated as follows:

(26) $$\bar{I}\left(t_{\mathrm{day}}\right)=\sum_{k=1}^{n}w_{k}I\left(t_{\mathrm{day}}-k\right),\sum_{k=1}^{n}w_{k}=1,$$

where n=11 and the coefficients ($w_{k}$) are shown in Figure 9b. It has a large weight in a small *k* and it has a small peak at k=7. We considered it a weekly period, and it almost converged to 0 around k=11. Comparing $I(t_{\mathrm{day}})$ and $\bar{I}\left(t_{\mathrm{day}}\right)$ (Figure 9a), we found that sudden peaks are reduced in the case of $\bar{I}\left(t_{\mathrm{day}}\right)$, and it fluctuates around the monthly economic index (CI).

## 3. Discussion

In this section, we summarize our method. First, we systematically found the words from many candidates (1352 words) that had strong correlations with the composite index (CI) coincidence by using Fisher’s exact test which is a non-parametric method and has robustness for outliers. We then applied grouping and round robin (detection of spurious correlation) to reduce the number of candidates which had a strong correlation with other candidates or a spurious correlation; As a result, we obtained 17 representative words. We graphically demonstrated the relationships between the words which helped to obtain an intuitive understanding of the results of the variable selections. We then proposed the regression method for the time series, which widely fluctuated. Our model reasonably reproduced the economic index using seven representative words which are statistically determined by the proposed method. We also showed the daily index by applying the regression results and the optimal moving average method. The announcement of an economic index by the government usually delayed because of the time required to gather and analyze data. The proposed method could reduce this time lag, because it is possible to collect blogs and calculate the index everyday.

The proposed method could be applied not only in estimating economic indicators but also in situations where we find good explanatory values to illustrate an explained value, and it is one of the most important tasks in analyzing big data.

## Figures and Tables

**Table 1:** Top 4 smallest *p*-value words with the composite index (CI).

Words	*p*-Value
Recession	$9.23\times 10^{-16}$
Stock Investment	$9.23\times 10^{-16}$
Commercial Bank	$4.77\times 10^{-14}$
IPO	$4.77\times 10^{-14}$

**Table 2:** A part of round robin results of P(i:j).

	*j*	Recession	Foreign Banks	Medical Expenses	Economic Measures
*i*					
**Recession**		—	39.2	31.4	31.7
**Foreign banks**		21.8	—	18.8	21.3
**Medical expenses**		9.4	11.6	—	15.0
**Economic measures**		7.6	12.8	13.5	—

**Table 3:** The regression coefficients ($c_{k}$) and intercept.

Word	Coefficients ($c_{k}$)
Recession	$-1.09\times 10^{4}$
Foreign banks	$2.07\times 10^{5}$
Primary balance	$2.70\times 10^{5}$
Allowance	$4.74\times 10^{3}$
Medical expenses	$1.50\times 10^{4}$
Long-term prime rate	$1.77\times 10^{5}$
Home sales	$4.21\times 10^{5}$
(intercept)	93.1
